# The Development of a Low-Cost Fresnel Lens UV Telescope with SiPM Array for Low-Light Atmospheric Transient Detection

**DOI:** 10.3390/s26072149

**Published:** 2026-03-31

**Authors:** Gabriel Chiritoi, Eugeniu Mihnea Popescu

**Affiliations:** Institute of Space Science—INFLPR Subsidiary, 409 Atomistilor, 077125 Magurele, Romania; empopescu@spacescience.ro

**Keywords:** dark matter, meteors, TLE, nuclearites, silicon photomultipliers, MPPC, front-end electronics, electronics, low-light detectors, single photon detector

## Abstract

This work presents the development and experimental characterization of a compact ultraviolet (UV) telescope based on silicon photomultipliers (SiPMs) designed for the detection of faint atmospheric optical tracks. Such transient optical phenomena include meteors, transient luminous events (TLEs), space debris reentries, and other faint atmospheric emissions. Nuclearite-induced atmospheric emission is considered as a benchmark case for evaluating the expected signal levels of rare luminous track events. We detail the fabrication, assembly, and testing of the SiPM sensor array, comprising parallel Geiger-mode avalanche diodes with high fill factor and photon detection efficiency, alongside custom readout electronics using self-triggering ASICs, precision optical components, and a stable mechanical mount. This photon-counting telescope provides a compact and mechanically robust alternative to conventional PMT-based systems, with demonstrated capability for detecting low-light atmospheric tracks under controlled laboratory conditions.

## 1. Introduction

Wide-field optical instruments capable of detecting faint atmospheric light emissions are essential tools for studying a variety of transient phenomena occurring in the Earth’s atmosphere. These include meteors produced by small interplanetary particles entering the atmosphere, transient luminous events (TLEs), such as sprites and elves associated with lightning activity, and optical signatures produced by re-entering space debris. Many of these phenomena generate faint ultraviolet or visible emissions that propagate over large distances and can be detected by sensitive optical detectors equipped with wide fields of view. Monitoring such events contributes to atmospheric science, space situational awareness, and the study of high-energy processes occurring in the upper atmosphere.

Recent advances in SiPM technology have enabled the development of compact photon-counting detectors with high sensitivity in the ultraviolet and visible spectral ranges. Compared to photomultiplier tubes (PMTs), SiPM devices offer advantages such as low operating voltage, mechanical robustness, compact size, and the possibility of assembling large multi-pixel arrays. These properties make SiPM-based systems attractive candidates for the development of distributed networks of compact optical telescopes designed to detect faint atmospheric transient events. However, the relatively high dark count rate of SiPMs introduces challenges for low-light detection and requires careful optimization of optical design, signal processing, and trigger strategies.

In this work, we present the design, construction, and experimental characterization of a compact ultraviolet telescope based on a multi-channel SiPM detector array. The primary goal of the instrument is to explore the feasibility of detecting faint atmospheric optical tracks using a compact and low-cost optical system. Particular emphasis is placed on the characterization of the optical response, photon detection efficiency, dark count rate, and signal processing chain of the detector. The instrument is therefore intended primarily as a prototype platform for the evaluation of the performance of SiPM-based ultraviolet telescopes for atmospheric transient detection.

Among the possible sources of faint atmospheric light tracks, hypothetical nuclearites have been proposed to produce continuous optical emission during their passage through the atmosphere. Although the main objective of the present work is not to perform a dedicated nuclearite search, nuclearite luminosity models provide a convenient benchmark for estimating the detectability of extremely faint track-like signals. In this context, the nuclearite case serves as a useful reference scenario for evaluating the sensitivity of the prototype detector to rare atmospheric events producing low photon fluxes.

Nuclearites [1] and similar exotic particles [2] have been the subject of various experiments employing different approaches. These experiments can be categorized based on detector characteristics, such as detection area, and particle properties, like minimum nuclearite mass or speed. The detection techniques can be summarized as follows: acoustic emission using aluminum alloy cylindrical detectors, tracks etched in plastic materials like CR-39, Makrofol, or Lexan, seismic waves induced by large nuclearites, and light emission in oil, seawater, or liquid xenon environments [3]. These techniques have been employed in several experiments, including the MACRO [4] underground detector at the Gran Sasso Laboratory in Italy, the SLIM [5] experiment (operated for over 4 years at the high-altitude Chacaltaya laboratory in Bolivia), the ANTARES telescope [6], and KM3NeT [7].

For an atmospheric nuclearite, De Rujula and Glashow [8] affirmed that during its motion through a continuous medium such as the atmospheric air or water, it produces a cylindrical expanding shockwave, the energy lost due to drag effects being essentially transferred to the surrounding medium, which heats up and emits blackbody radiation. The estimated visual magnitude at a distance, h, from the observer:

(1) $$M=10.8-1.67\log_{10}\left(\frac{m}{1\;\mu g}\right)+5\times \log_{10}\left(\frac{h}{10\;\mathrm{km}}\right),$$

where *m* is the mass of the nuclearite. In contrast to meteors, which generally emit light in the upper atmosphere and travel at approximately 72 km/s, the upper limit of the altitude where nuclearites can emit light *h_max_* is calculated with the formula:

(2) hmax=2.7kmln(m1.2×10−5 g),

Nuclearites exhibit luminosity approximately proportional to atmospheric density, with most light emitted near ground level. Due to their compact size, nuclearites heavier than 10^−4^ g can penetrate the atmosphere, and those above 0.1 g may traverse the Earth entirely. Additionally, upward trajectories are possible for sufficiently massive candidates. These characteristics provide discriminating features that distinguish nuclearites from meteors [9,10,11]. These super-heavy particles emitted radiation can be detected using the method developed for ultra-high-energy cosmic rays: observing their atmospheric UV fluorescence and Cherenkov light with dedicated terrestrial or orbital UV telescopes. However, despite extensive searches by projects such as “Pi of the Sky” [11] and bar detectors like Nautilus [9], direct evidence for nuclearites remains elusive, leading to stringent flux limits that constrain their abundance in the universe [10,11].

**Figure 1:**
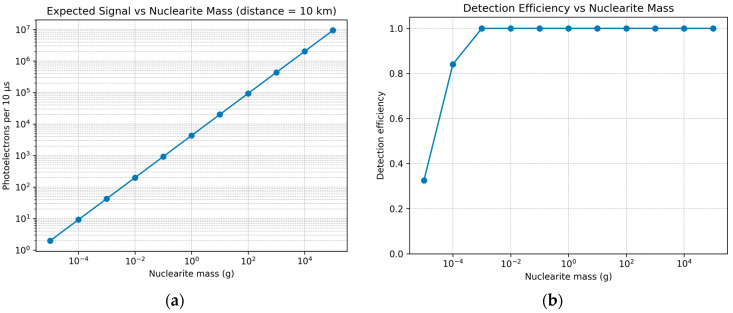
(**a**) Expected signal at the detector for nuclearites traveling at 250 km/s at a 10 km distance; (**b**) detection efficiency vs. nuclearite mass.

In Figure 1, we represented our instrument detection efficiency as a function of nuclearite mass, which was estimated via a Monte Carlo approach following the methodology commonly used in wide-field optical searches (e.g., Pi of the Sky [11]). For each mass bin, nuclearite trajectories were generated isotropically, and they were intersected with the telescope field-of-view cone and the luminous altitude range [0, *h_max_*(m)], where *h_max_* was calculated from the nuclearite mass using an exponential atmosphere parametrization. The nuclearite luminosity model is used here to estimate the minimum photon flux required for the detection of faint atmospheric tracks. The efficiency curve represents a first-order detectability estimate based on geometric acceptance and signal-level assumptions rather than a full end-to-end sensitivity calculation including firmware trigger performance and measured false trigger rates.

For each visible track segment, the apparent magnitude along the trajectory was computed using a De Rujula–Glashow type model and converted into an expected photoelectron yield per integration window, Δ*t* = 10 μs, using the telescope aperture (*D* = 254 mm), total optical throughput (*η_opt_* ≈ 0.70), and SiPM photon detection efficiency. The triggering was evaluated using a coincidence criterion requiring simultaneous multi-photoelectron signals in adjacent pixels persisting for multiple consecutive windows, suppressing random dark count triggers. The detection efficiency, *ϵ*(*m*), was defined as the fraction of simulated nuclearites producing an observable track segment that satisfied the trigger criteria.

Similar optical concepts employing Fresnel lenses and multi-pixel photon [12,13,14] detectors have been implemented in fluorescence-based cosmic ray observatories, including the FAMOUS [15] prototype and the EUSO/Mini-EUSO missions [16]. These systems demonstrate the feasibility of wide-field UV detection of atmospheric phenomena using lightweight refractive optics. The present work does not introduce a fundamentally new detection principle; rather, it explores a compact, ground-deployable and cost-efficient implementation based on commercially available SiPM arrays and self-triggering ASIC readout electronics. This work should be viewed as complementary to existing instruments, demonstrating that SiPM technology can be adapted for extremely low-cost, portable telescopes suitable for multi-unit arrays, educational purposes, and niche scientific applications where large facilities are not cost-effective.

The document is structured as follows: Section 2 briefly presents the operational parameters relevant to SiPMs and front-end electronics; Section 3 discusses the assembly and testing of the SiPM camera; and Section 4 concludes with a summary of our results.

## 2. Materials and Methods

### 2.1. SiPM Characterization Setup

Silicon photomultipliers (SiPMs) are solid-state photon detectors comprising arrays of single-photon avalanche diodes (SPADs) operating in Geiger mode above the breakdown voltage [17]. Each SPAD produces a standardized avalanche pulse upon photon detection, and the summed output provides a signal proportional to the number of detected photons. SiPMs offer advantages over conventional photomultiplier tubes, including compact form factor, insensitivity to magnetic fields, and low operating voltage (~50–60 V). For detailed reviews of SiPM technology, see [17,18,19].

**Figure 2:**
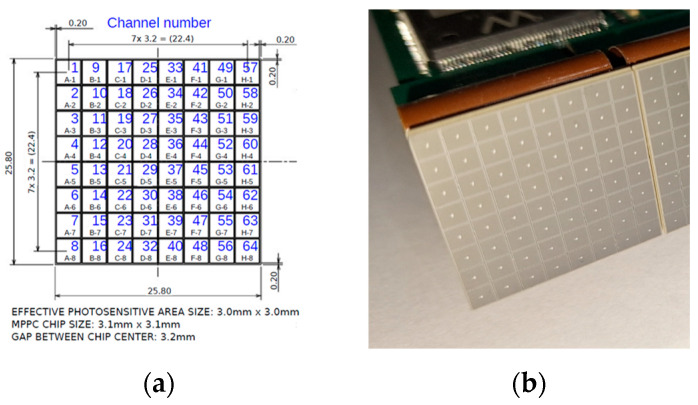
Hamamatsu S13361-3050AE-08 SiPM: (**a**) diagram and physical dimension; (**b**) a picture of the SiPM matrix.

To meet the requirements for our single-photon detector, we evaluated several SiPMs from different manufacturers and ultimately selected the Hamamatsu S13361-3050AE-08 (Hamamatsu Photonics, Chuo-ku, Hamamatsu City, Shizuoka Pref., Japan) [20]. This SiPM matrix consists of 64 Through-Silicon-Via SiPM channels arranged in an 8 × 8 configuration, with each channel containing 3584 Geiger avalanche photodiodes at a 50 μm pitch. Figure 2 illustrates the layout of the 64 channels and the array’s physical dimensions of 25.8 mm × 25.8 mm. The SiPM matrix has an overall fill factor of 74%. Its surface is coated with a silicon resin (refractive index of 1.55), enabling detection of wavelengths down to 270 nm. For connectivity, the detector is provided with SAMTEC ST4-40-1.00-L-D-P-T connectors (Samtec Inc., New Albany, IN, USA) on the backside. Some electrical and optical characteristics of the Hamamatsu S13361-3050AE-08 at T = 25 °C and ΔV = 3 V are detailed in Table 1 [20].

The SiPM characterization was performed in a temperature-controlled environment maintained at 25 ± 0.2 °C to minimize the effects of temperature variations on dark count rate and gain during calibration.

The bias voltage was supplied by a precision programmable source with stability better than 1 mV (Rohde & Schwarz HMP2030-Rohde & Schwarz, Munich, Germany), ensuring an accurate determination of the breakdown voltage and of the gain as a function of the overvoltage.

The dark count rate (DCR) measurements were carried out using a digital oscilloscope in the time-tag mode (Tektronix MSO 5204B–Tektronix Inc., Beaverton, OR, USA), recording time intervals between consecutive pulses to build a statistical distribution of dark events, and with CAEN A5202 DAQ (CAEN SpA, Viareggio (LU), Italy).

The CAEN A5202 acquisition chain consisted of a 14-bit ADC operating at 65 MS/s with an adjustable discriminator threshold corresponding to approximately 0.3 photoelectrons (p.e.).

The acquisition window was set to 10 μs, allowing the analysis of both fast single-photon signals and delayed after-pulsing phenomena.

Gain and photon detection efficiency (PDE) calibrations were performed using a pulsed ultraviolet LED (λ = 365 nm from Vishay, manufacturer model VLMU1610-365-135 (Vishay Intertechnology Inc., Malvern, PA, USA) [21]) driven by a function generator (Keysight 33600A-Keysight Technologies Inc., Santa Rosa, CA, USA).

The absolute photon flux was determined using a NIST-traceable silicon photodiode (Ophir PD300-UV-Ophir Optronics Solutions, Jerusalem, Israel) to obtain a reference optical power.

The PDE was calculated by comparing the detected p.e. count rate to the incident photon rate over a bias voltage range of 53–56 V, covering several overvoltage points above breakdown.

### 2.2. Front-End Electronics and Signal Processing

The SiPM array was interfaced with a CAEN A5202 [22] data acquisition system incorporating two Citiroc-1A ASICs (Weeroc, Villebon-sur Yvette, France) [23].

**Figure 3:**
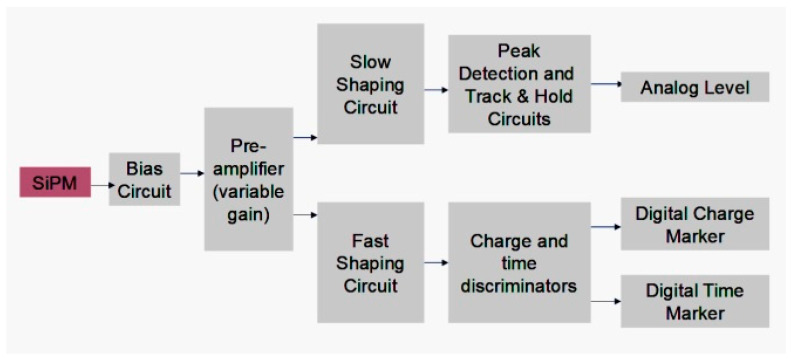
Functional schematic of the Citiroc 1A readout ASIC.

Each ASIC channel includes two shaping chains: a fast path (shaping constant 50 ns) for timing discrimination and a slow path (shaping constant 150 ns) for charge integration (Figure 3).

The programmable preamplifier gain was tuned to achieve a conversion factor of approximately 1 mV/p.e., balancing linearity and dynamic range.

The fast-shaping chain output of each CITIROC channel is connected to the internal time discriminator. The leading-edge crossing of this discriminator provides a timing signal for each channel. The FPGA records these timestamps and performs the coincidence analysis across neighboring pixels. The Time-over-Threshold (ToT) measurement implemented in the FPGA is used as an estimate of pulse duration and deposited charge, while the timing information refers specifically to the discriminator leading-edge crossing times. This approach enables both temporal and spatial clustering of signals to suppress random dark-count triggers. The FPGA also managed data buffering and trigger synchronization between channels. The waveforms were stored via the CAEN control (v5203) software and analyzed offline using a custom Python (v3.14) interface for digital signal reconstruction, baseline subtraction, and photoelectron counting.

### 2.3. Optical System Characterization

**Figure 4:**
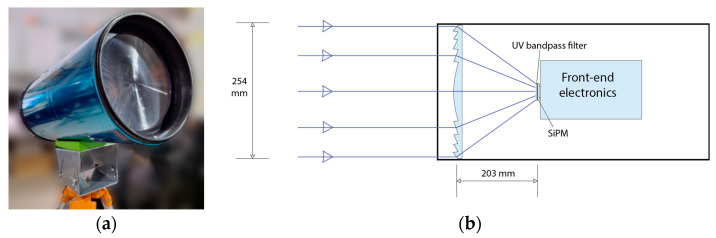
Optical setup: (**a**) telescope system; (**b**) schematic drawing of the optical design.

From a mechanical design perspective, aimed at achieving portability and full-sky coverage for detecting fast-moving atmospheric light tracks, we fabricated a cylindrical telescope body mounted on a rotational Alt-Azimuth pod. This configuration allows the field of view to be pointed freely upwards or downwards, facilitating the observations from zenith to horizon. Inside the body, we integrated the optical components with the SiPM camera at the focal plane (Figure 4a). The main optics consist of an ultraviolet-transmitting acrylic Fresnel lens from Fresnel Technologies [24], with a 254 mm diameter, 203 mm focal length (yielding a fast f-number of f/0.8), and 90% transmittance in the UV region. The low f-number maximizes photon collection efficiency from faint events, while the lightweight acrylic construction—significantly lighter than equivalent glass lenses—enables the deployment in remote locations without heavy infrastructure or support. In front of the SiPM, we mounted a Thorlabs FGUV5S bandpass UV filter (Thorlabs, Newton, NJ, USA) [25] to restrict the spectral range of incident light and to suppress visible sky glow and artificial illumination. Although the Hamamatsu S13361-3050AE-08 SiPM exhibits peak sensitivity near 450 nm, it retains significant sensitivity in the near-UV; therefore, the selected 320–395 nm bandpass is chosen to improve signal-to-background for UV-dominant atmospheric emission while reducing sensitivity to broadband visible backgrounds. The schematic drawing of the optical design is illustrated in Figure 4b.

The telescope’s total field of view is approximately 8°, corresponding to ~1° per channel of the 8 × 8 sensor array. These parameters imply that, for an exotic particle (e.g., nuclearite), interacting with the atmosphere at 10 km altitude and traveling at v = 250 km/s (assuming overhead passage with horizontal velocity component dominating), the telescope can record its light tracks for a duration of t ≈ 5 ms; at 50 km altitude, this extends to t ≈ 28 ms.

Optical characterization of the telescope was performed using a calibrated Ocean Optics HR4000 spectrometer (Ocean Optics Inc., Orlando, FL, USA).

**Figure 5:**
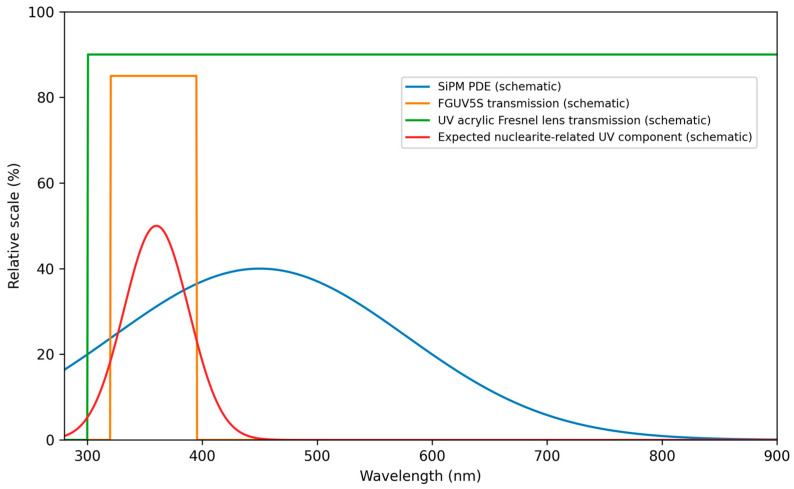
Schematic spectral overlap of the SiPM response.

The transmittance spectra of both the UV-transmitting Fresnel lens (Fresnel Technologies Inc., Fort Worth, TX, USA) and the Thorlabs FGUV5S bandpass filter (Thorlabs, Newton, NJ, USA) were measured between 300 nm and 400 nm. The Fresnel lens exhibited an average transmittance of 89.3% at 365 nm, while the FGUV5S filter maintained 81.6% average transmittance across its 320–395 nm passband; an overlapping optical transmission of the system components is illustrated in Figure 5.

The optical focal length was verified experimentally using a collimated UV beam directed through the lens onto a CMOS detector (Thorlabs, Newton, NJ, USA) positioned at the focal plane.

The measured focal length was 203 ± 3 mm, consistent with design specifications, and the optical modeling (performed in Lambda Research Oslo (v22.1) and TracePro (v24.3)) indicated a spot size of <1 mm RMS at the focal plane throughout the 8° field of view (FOV).

The overall optical throughput was estimated at ~70% after accounting for all optical losses.

### 2.4. Dark Chamber and Light Shower Tests

**Figure 6:**
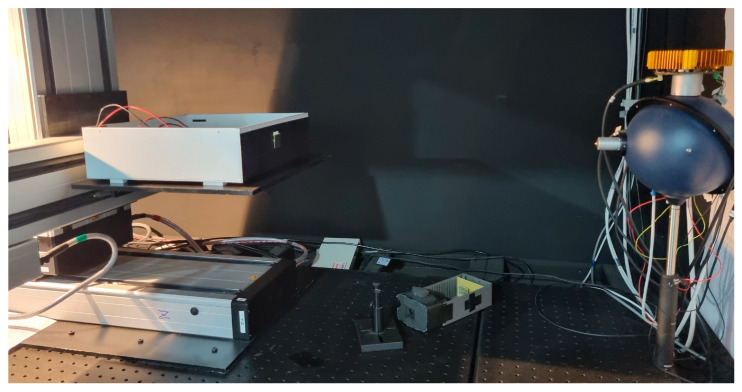
Dark chamber facility.

All system tests were performed inside a dark chamber facility designed for low-background optical measurements—a light-tight box equipped with various sensors, light sources, and an XYZ stage for micrometric position, as illustrated in Figure 6.

For pixel-by-pixel characterization, a uniform light source was used by means of an integrated sphere coated internally with SPECTRALON (for maximum UV reflectance). One port of the integrated sphere was equipped with an Ophir NIST calibrated UV sensor for power measurement, in another port is fed one UV LED light source [21] centered at 365 nm while the third port of 1.5 mm diameter act as exit light port. The SiPM was placed at 50 cm distance on a plate connected to an XYZ stage. In order to control the duration, period, and amplitude of the UV pulses, a function generator model, Keysight 33600A (Keysight Technologies Inc., Santa Rosa, CA, USA), was used, providing the UV optic pulses with width of 20 ns and with a period of 1µs. The illumination level for one pulse provides an average of 15 photons for each SiPM cell in the matrix. In order to allow single-photon counting, the external trigger from the function generator was synchronized with the DAQ system.

Absolute light intensity was verified using an Ophir NIST-calibrated power meter, and the photon flux was computed according to:

(3) $$N_{ph,pulse}=\frac{P_{light}}{E_{ph}},$$

where *E_ph_* = hν is the photon energy at 365 nm.

Environmental parameters—including temperature, humidity, and residual background light—were continuously logged to ensure repeatability.

## 3. Results and Measurements

### 3.1. Gain and Breakdown Voltage Measurement

The gain of an MPPC is the factor by which a Geiger-mode avalanche multiplies the initiating electron; it is proportional to the overvoltage (*V_over_*), defined as the difference between the applied bias voltage and the device’s inherent breakdown voltage (*V_B__R_*). The Geiger-mode avalanche can be initiated either by the photoelectric effect or by thermal carrier excitation.

A direct method to measure *V_BR_* is obtained from the *x*-axis intercept of an MPPC gain versus bias voltage plot. Assuming a proportionality factor, k, between gain and overvoltage, the gain (*G*) is given by the following equation:

(4) $$G=k\times V_{over}=k\times (V_{bias}-V_{BR}),$$

where when *G* = 0, the value of *V_bias_* matches the breakdown voltage *V_BR_*.

Calculating *V_BR_* is necessary for other measurements that rely on the overvoltage, *V_over_*. Moreover, *V_BR_* varies from one MPPC to another within the array, even though they are part of the same solid-state device. Consequently, it is recommended to characterize and calibrate the properties of the MPPCs within the same array or across different arrays that comprise a larger device.

The CITIROC UI application provides histograms of the Slow Shaper outputs, with amplitudes proportional to the signals generated by the MPPCs in the 8 × 8 matrix. Each histogram represents a series of digitized values proportional to the MPPC’s output charge corresponding to the pulse count.

**Figure 7:**
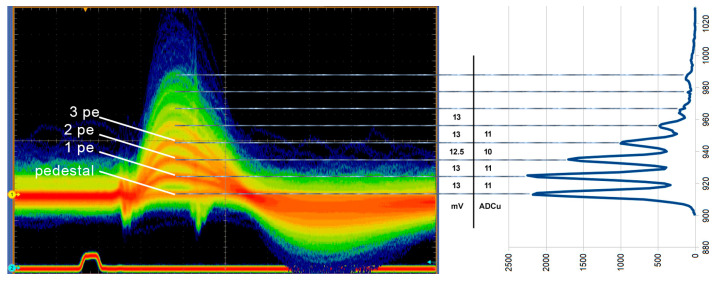
Distinctive 1 p.e., 2 p.e., signals measured with an oscilloscope, and the corresponding pulse count histogram at *V_bias_* = 54.0 V.

The persistent display mode superimposes the various p.e. levels of the Slow Shaper output, ranging from the noise pedestal baseline to the consecutive higher levels corresponding to 1 p.e., 2 p.e., and so on. These levels are proportional to the SiPM pulse heights, as shown in Figure 7. Various Persistent Display Views of the Slow Shaper Output have been captured with the oscilloscope, starting from *V_bias_* = 51.6 V up to 55.0 V, in steps of 0.2 V, for channel (SiPM) #20 (one of the SiPM cells placed in the center of the matrix), as shown in Figure 7.

**Figure 8:**
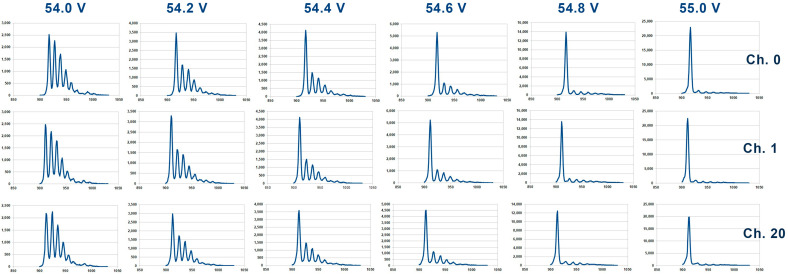
Preliminary results from the characterization of the SiPM detector show the finger plot for 3 channels (0, 1, and 20) for different bias voltage.

Using the data collected above for each of the 64 channels, a number of six histograms (or “finger diagrams”) can be drawn, one for each *V_bias_* value. Figure 8 presents some of these histograms, for channel 0, channel 1, and channel 20. From these histograms, it can be remarked the presence of several significant peak levels corresponding to the pedestal and several p.e. levels. Also, it can be remarked the constant increase in the pedestal level (noise) with the rise in *V_bias_* level, to a level which is significantly larger at *V_bias_* = 55.0 V and 54.8 V than the other p.e. levels for the lower voltages. In each histogram, the value of the gap between two consecutive p.e. peaks represent the value of the gain of the SiPM measured in ADU. The gaps are “widening” with the increase in *V_bias_*. Theoretically, the value of the gaps in the same histogram should be equal, but slight differences are present, and therefore in order to calculate the most accurate value of the gain, a mean value of the gap value has been determined. Regardless of the amplitude of the pedestal levels, the first five gaps starting with the pedestal level have been considered for the mean gap value calculation, which is in fact the value of the gain.

**Figure 9:**
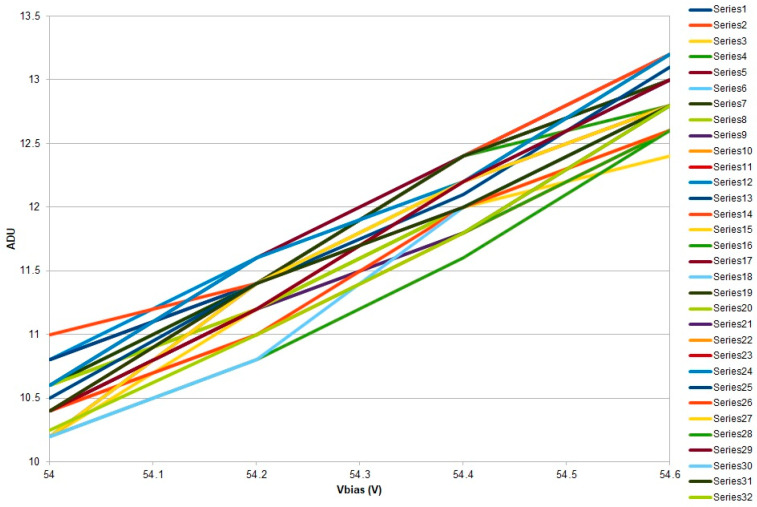
*Gain* (in ADU units) vs. *V_bias_* plot for bias voltages from 54.0 V to 54.6 V across 32 channels.

The gain calculations for all 64 channels at *V_bias_* = 54.0 V to 55.0 V have been performed, supplying the data for a *gain* vs. *V_bias_* plot. The resulting plot undergoes linear fitting, with the *x*-axis intercept yielding the breakdown voltage, *V_BR_*, of the tested MPPC. An example of such a gain plot, in ADU units, for bias voltages from 54.0 V to 54.6 V across only 32 channels (to maintain figure clarity) is shown in Figure 9.

We can conclude that the SiPM gain exhibited a linear dependence on applied bias voltage. The channel-to-channel variation across the 64 elements of the SiPM array was below 1.8%, demonstrating high uniformity.

The linear regression yielded R^2^ = 0.998, confirming excellent linear behavior in the operating region.

By determining the intercept point for each of the graphs with the *x*-axis (*V_bias_* axis), the following results representing the breakdown voltages are obtained in Table 2, where it can be observed that the SiPM cells in the matrix differ from the point of view of *V_br_*, in a range of 0.587 V between 51.465 V and 52.043 V:

### 3.2. Photon Counting and PDE Measurement

To characterize single-photoelectron (1 p.e.) resolution, we measured the charge spectrum under very low illumination conditions. By using external trigger synchronized to LED pulses, we eliminate the DCR background and measure only the response to known light pulses, allowing clear discrimination of 1, 2, 3, 4, and 5 photoelectron events (Figure 7).

For continuous self-triggered operation, isolated single-pixel signals at the level of 1–5 photoelectrons are not sufficient because of the intrinsic dark count rate of SiPM detectors. In theory, a 1 g nuclearite traversing the atmosphere at 250 km/s at 25 km altitude generates observable tracks lasting 5–15 ms depending on the simulated altitude (10–50 km) and an estimated flux level of ~3 × 10^7^ photons/m^2^/s at pupil entrance. This can be translated, after introducing the aperture area, the optical throughput, and PDE, to a detected photoelectron rate of about ~4 × 10^5^ p.e./s or 4 p.e. (entire detector) per integration window of 10 µs. Our trigger proposed using spatial coincidence (≥3 adjacent channels) to suppress random dark count triggers. In this case, the signal-to-noise analysis (SNR_3ch_ ≈ 0.31) reveals fundamental limitations of this approach at the 10 μs timescale and insufficient for reliable detection.

Therefore, the trigger strategy adopted for the present prototype is based on spatio-temporal coincidence rather than single-channel amplitude alone. In this configuration, a trigger candidate is formed when at least three adjacent pixels exceed a threshold of approximately 4–5 photoelectrons within the same 10 μs integration window, with persistence over at least three consecutive windows. Candidate events are subsequently validated by requiring millisecond-scale temporal clustering and coherent track-like evolution across neighboring pixels as the luminous source traverses the field of view. Because thermal dark counts are uncorrelated in both space and time, the probability of producing such multi-pixel, multi-window patterns randomly is extremely small. This topology-based trigger concept is similar to the pattern-recognition techniques employed in wide-field optical transient searches and fluorescence telescopes to discriminate real atmospheric events from detector noise inspired by Mini-EUSO’s multi-level trigger [16], which operates at 2.5 µs (photon counting), 320 µs (transient detection), and 40.96 ms (continuous acquisition) timescales. Our longer fundamental integration window (10 µs vs. 2.5 µs) reflects the trade-off between readout complexity and temporal resolution appropriate for millisecond-duration atmospheric tracks.

However, in order to approach this stringent limit, a set of required improvements for viable detection can be assessed in the future: cooling to −15 °C (4× DCR reduction), lager aperture or detector area (a 500 mm aperture-3.9× signal increase), higher PDE SiPMs (1.6× signal increase), and optimized FPGA trigger logic.

Due to fundamental SNR limitations at the required integration timescales described above, the instrument is well-suited for brighter atmospheric transients where signal exceeds DCR by a comfortable margin:

1. Meteors:
  - Typical magnitude: −2 to +2 (brighter than nuclearites by 10^3^–10^6^×).
  - Expected signal: 10^7^–10^9^ p.e./s.
  - SNR (10 μs window): 100–1000 (easily detectable).
  - Clearly detectable.
2. Lightning sprites:
  - Typical luminosity: 10^4^–10^7^× brighter than nuclearites.
  - Expected signal: 10^9^–10^12^ p.e./s.
  - SNR (10 μs window): 1000–10,000.
  - Status: Clearly detectable.
3. Satellite flares:
  - Typical magnitude: −5 to +2.
  - Expected signal: 10^6^–10^9^ p.e./s.
  - SNR (10 μs window): 10–1000.
  - Detectable to clearly detectable.

We characterized the intrinsic PDE of the bare SiPM sensor at 450 nm to compare with manufacturer specifications and validate our measurement setup. The measured photon detection efficiency (PDE) was 37.8 ± 1.2% at 450 nm, with stable response over the full active area. This is a confirmation that system-level optical throughput (300–400 nm) is assured in terms of PDE, the slight deviation being attributed to experimental factors.

Flat-field illumination tests yielded >92% pixel-to-pixel uniformity, demonstrating high spatial homogeneity across the 8 × 8 array.

### 3.3. Dark Count, After-Pulsing, and Crosstalk

**Figure 10:**
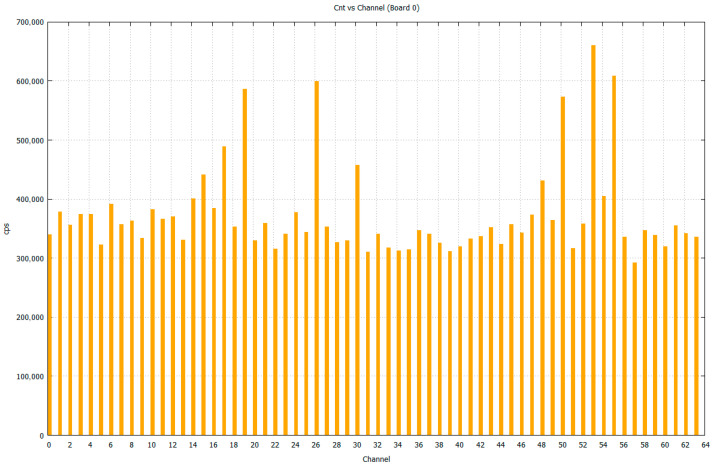
The DCR of the 64-channel SiPM array inside the dark chamber at 25 °C without any external light input, *V_bias_* 54.6 V.

As is illustrated in Figure 10, the dark count rate (DCR) was measured at ~0.4 Mcps per pixel per second in the dark chamber light-tight environment at 25 °C and approximately doubled for every 10 °C increase in temperature. For field deployment in uncontrolled environments, we plan to combine a passive thermal mass and a weather-sealed enclosure, real-time temperature monitoring, and bias-voltage compensation using the device temperature coefficient (Δ*V_op_*/Δ*T*) to keep the overvoltage approximately constant. In addition, adaptive trigger thresholds can be adjusted in firmware to maintain a stable false-trigger rate under varying temperature conditions.

Optical crosstalk probability was determined to be ~3% using coincidence timing analysis, while the after-pulsing fraction remained below 1%.

These low correlated noise values confirm that the SiPM array is suitable for single-photon-level detection in low-background environments.

### 3.4. Optical and Angular Response

**Figure 11:**
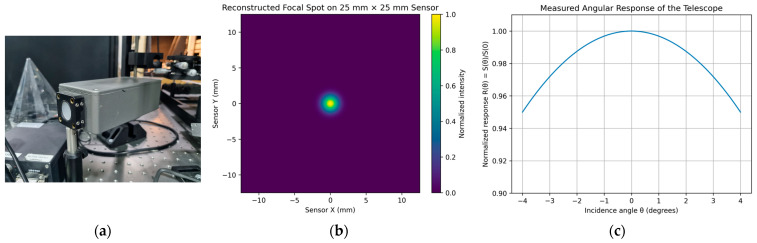
Optical and angular response: (**a**) Collimated UV source at 365 nm, comprising a matrix of 3 × 3 LEDs [19] inside an integrated sphere provided with a diffuser at the output port, which generate a uniform spot with a 40 mm beam diameter at the Fresnel lens entrance. (**b**) reconstructed focal spot distribution across the 25 mm × 25 mm SiPM sensor plane obtained from the focal plane scanning measurement. (**c**) Measured angular response of the telescope. The detector signal, *S*(*θ*), was normalized to the on-axis response, *S*(0). The response remains within ~5% of the on-axis value across the ±4° field of view.

Optical response was characterized using a collimated UV source at 365 nm, comprising a matrix of 3 × 3 LEDs [21] inside an integrated sphere provided with a diffuser at the output port, which generate a uniform spot with 40 mm beam diameter at the Fresnel lens entrance, placed on a rotational stage for the angular measurements, as shown in Figure 11a. The total throughput was obtained from the ratio of detected to incident optical power, while focal plane spot quality was reconstructed by scanning the detector across the focal plane and computing the RMS spot radius from the measured 2D intensity distribution. Angular response, *R*, was measured by varying the incidence angle, *θ*, of the collimated beam with respect to the optical axis and normalizing the integrated detector signal, *S*(*θ*), to the on-axis response, *S*(0), yielding *R*(*θ*) = *S*(*θ*)/*S*(0). The above measurements are illustrated in Figure 11.

The Fresnel lens and bandpass filter combination maintained transmission efficiencies of 89.3% and 81.6%, respectively, and provides an RMS spot radius at ~1 mm, which indicates that most of the ray energy is concentrated within a region significantly smaller than the detector pixel pitch, ensuring minimal optical blur and efficient coupling of incident photons to individual pixels across the full 8° field of view, and the angular response remained within 95% of the on-axis value up to ±4°.

Angular response measurements showed less than 5% attenuation up to ±4° off-axis, consistent with the simulated FOV.

The uniform angular response ensures accurate photon collection across the detector plane, minimizing edge losses.

### 3.5. Simulated Event

To evaluate the response of the prototype telescope to faint, spatially extended optical transients, we developed a laboratory light shower emulator based on a pulsed ultraviolet LED array. The emulator was not intended to reproduce all physical aspects of a real atmospheric event but rather to generate controlled low-light signals with millisecond-scale duration and photon flux levels comparable to those expected from very faint track-like atmospheric sources. In this sense, the setup provides a bench-top validation of detector response and trigger concepts rather than a direct demonstration of nuclearite detectability. The emulator consisted of a compact array of 15 UV LEDs centered at 365 nm, mounted on a common printed circuit board, and driven by a programmable pulse generator. The source was positioned approximately 5 m on-axis from the telescope aperture and was coupled to a diffuser in order to illuminate the entrance pupil with a reproducible low-intensity extended signal. The pulse width and repetition parameters were adjusted to generate optical pulses in the millisecond regime, consistent with the characteristic duration expected for faint atmospheric tracks crossing the field of view of the telescope. The intensity of the emulator was calibrated using the same optical power measurement chain employed in the detector characterization, allowing the conversion of the emitted optical power into an estimated photon flux at the telescope entrance. In the benchmark configuration used in this work, the source intensity was adjusted to reproduce a photon flux of the order of 3 × 10^7^ photons/m^2^/s at the pupil entrance, corresponding to the order of magnitude expected from a very faint atmospheric track under the simplified luminosity model discussed in Section 3.2. This benchmark level was chosen because it lies close to the low-signal regime where detector noise and trigger topology become critical.

The resulting detector response was analyzed in terms of spatial hit pattern, temporal persistence, and integrated signal across neighboring channels by using the trigger of 320 µs (transient detection) timescale. As discussed in Section 3.2, the measured dark count rate of the SiPM array prevents reliable self-triggering on isolated low-amplitude single-pixel signals. Therefore, the purpose of these emulation measurements was to verify that faint optical pulses distributed across multiple adjacent channels can still be identified through spatio-temporal correlation rather than through the instantaneous single-channel amplitude alone. In particular, the measurements were used to validate the concept of combining short integration windows with track-like clustering across adjacent pixels over the full event duration.

**Figure 12:**
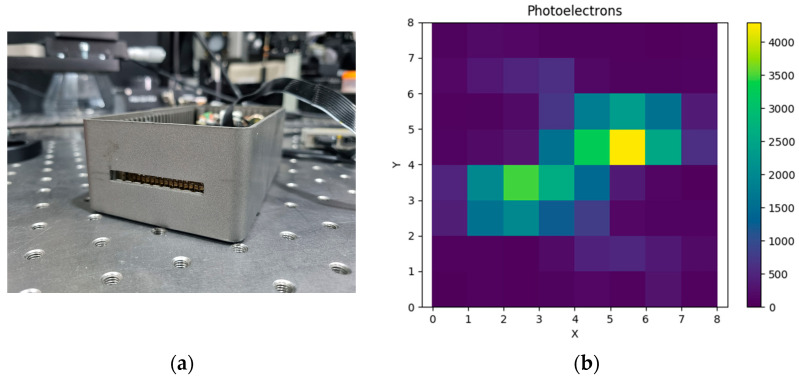
(**a**) Light shower emulator as an 15 UV LEDs array. (**b**) The response of the 8 × 8 SiPM array to a 10 ms pulse light shower simulator source, which mimics light produced at a 25 km altitude by a nuclearite traveling at 250 km/s at a 320 µs integration time window.

Figure 12b shows a representative detector response obtained with the light shower emulator. The measured event exhibits a coherent multi-pixel pattern extending over several integration windows, consistent with the expected behavior of a faint moving optical transient. These measurements demonstrate that the prototype is capable of recording and reconstructing low-light, millisecond-scale atmospheric track-like signals under controlled laboratory conditions. However, they should not be interpreted as a full end-to-end demonstration of nuclearite detection sensitivity, since realistic sky background, outdoor operating conditions, and final trigger firmware optimization remain to be investigated.

## 4. Conclusions

We have presented the development and laboratory characterization of a compact, low-cost SiPM-based UV telescope platform for multi-application atmospheric transient detection. The instrument demonstrates a photon-counting capability with 37.8% photon detection efficiency in the UV band, 8° field of view, and millisecond-scale temporal resolution suitable for detecting faint optical tracks from meteors, transient luminous events, space debris, and potentially exotic particles. We emphasize that this work presents laboratory characterization and theoretical feasibility analysis, not demonstrated field performance. The instrument’s utility for nuclearite detection remains uncertain and would require: (1) a detailed Monte Carlo simulation of trigger efficiency and false trigger rates, (2) field deployment with known calibration sources, (3) operation at a reduced temperature to mitigate dark count rate, (4) sophisticated multi-pixel coincidence algorithms, and (5) increasing the FOV of the instrument for a better sensitivity through the collection of more photons. Nuclearites should be considered a challenging benchmark target rather than a primary application.

## Figures and Tables

**Table 1:** Hamamatsu S13361-3050AE-08 SiPM characteristics.

Characteristic	Specification
Spectral response range (*λ*)	320 to 900 nm
Peak sensitivity wavelength (*λp*)	450 nm
Photon detection efficiency: *PDE*	40%, at λp
Dark count	0.5–1.5 Mcps
Terminal capacitance (*Ct*)	320 pF
Gain (*M*)	1.7 × 10^6^
Breakdown voltage	*V_br_* = 53 ± 5 V
Recommended operating voltage	*V_op_* = *V_br_* + 3 V
*V_op_* variation between channels	±0.05 ± 0.15 V
Temperature coefficient of *V_op_* (Δ*TV_op_*)	54 mV/°C

**Table 2:** Measured breakdown voltages for only 32 channels.

Channel	0	1	2	3	4	5	6	7
*V_br_* (V)	51,513	51,530	51,513	51,513	51,779	51,597	51,625	51,519
Channel	8	9	10	11	12	13	14	15
*V_br_* (V)	51,465	51,763	51,769	51,804	51,769	51,597	51,763	51,8
Channel	16	17	18	19	20	21	22	23
*V_br_* (V)	51,569	51,845	51,926	51,727	51,664	51,914	51,878	51,779
Channel	24	25	26	27	28	29	30	31
*V_br_* (V)	N/A	51,794	51,821	51,968	51,892	52,043	51,859	51,965

## Data Availability

The original contributions presented in this study are included in the article. Further inquiries can be directed to the corresponding author.
